# Immune-related adverse reactions across multiple systems in a non-small cell lung cancer patient induced by immune checkpoint inhibitors: a case report and literature review

**DOI:** 10.3389/fphar.2026.1943059

**Published:** 2026-10-23

**Authors:** Jing Li, Dan Wang, Lingli Zheng, Chaomin Liu, Ting Jiang

**Affiliations:** 1 Clinical Medical College, Chengdu Medical College, Chengdu, Sichuan, China; 2 Department of Pharmacy, The First Affiliated Hospital of Chengdu Medical College, Chengdu, Sichuan, China; 3 Department of Oncology, The First Affiliated Hospital of Chengdu Medical College, Chengdu, Sichuan, China

**Keywords:** case report, immune checkpoint inhibitors, immune-related adverse events, multiple systems, toripalimab

## Abstract

**Background:**

Immune checkpoint inhibitors (ICIs) have revolutionized the treatment of non-small cell lung cancer (NSCLC), while overlapping multi-system immune-related adverse events (irAEs) are life-threatening and pose great clinical challenges.

**Case Description:**

A 74-year-old male patient with stage IIIA lung squamous cell carcinoma developed bilateral ptosis, blurred vision, abnormal thyroid function, elevated muscle enzymes and cardiac biomarkers after two cycles of toripalimab combined with chemotherapy. He was diagnosed with ICI-induced myositis, myocarditis and thyroiditis. ICIs were discontinued immediately. Glucocorticoids, methimazole and levothyroxine were administered sequentially according to disease progression. The patient recovered well and underwent lobectomy successfully.

**Conclusion:**

Elderly individuals complicated with pre-existing cardiovascular diseases are susceptible to severe immune-related adverse events. Early identification of overlapping multi-system irAEs, timely drug withdrawal and standardized immunosuppressive therapy are essential. Endocrine-immune-related adverse reactions exhibit dynamic evolution; for instance, thyroid toxicity may progress from a hyperthyroid phase to hypothyroidism, necessitating individualized monitoring and replacement therapy according to different stages of thyroid function.

## 1 Introduction

According to the GLOBOCAN 2022 global cancer statistics report, lung cancer is the leading cause of both new cancer cases and cancer-related deaths worldwide, with 2.481 million new cases and 1.817 million deaths reported. It ranks third in both crude incidence and age-standardized incidence rates, at 31.5 per 100,000 and 23.6 per 100,000, respectively, and holds the highest position in both crude mortality and age-standardized mortality rates, at 23.1 per 100,000 and 16.8 per 100,000, respectively (Filho et al., 2024). These statistics underscore the substantial global burden of lung cancer. The advent of immune checkpoint inhibitors has revolutionized the therapeutic landscape for non-small cell lung cancer (NSCLC), establishing them as the standard first-line treatment for advanced NSCLC cases lacking driver mutations. These inhibitors have significantly enhanced overall survival rates and are now being utilized across various stages of the disease, ranging from later-line treatments for advanced cases to early-stage neoadjuvant, adjuvant, and locally advanced consolidation therapies (Riely et al., 2026). While immune checkpoint inhibitors are effective in targeting tumors, they can also lead to the overactivation of T cells, resulting in the attack of normal tissues and the onset of immune-related adverse events (irAEs). The timing of irAEs varies across different organ systems and can manifest at any stage of immunotherapy. Early diagnosis and prompt treatment can render most irAEs completely reversible; however, 0.5%–13% of cases may become life-threatening, with a median onset of 1 month and rapid progression (Chinese Working Committee of Chinese Society of Clinical Oncology CSCO, 2023). The occurrence of overlapping irAEs across multiple systems presents a significant clinical challenge during treatment with immune checkpoint inhibitors (ICIs). These overlapping adverse effects not only compromise patients’ quality of life but can also necessitate treatment interruptions and pose life-threatening risks. A major challenge faced by clinicians in managing overlapping irAEs across multiple systems is the absence of standardized diagnostic criteria and management guidelines. A study (Ye et al., 2024) of 646 cancer patients found that 9.4% developed multi-organ system immune-related adverse events (irAEs). Of those with multi-system irAEs, 52.5% had received combined immunotherapy and chemotherapy or targeted therapy. Combination chemotherapy was identified as an independent risk factor for multi-system irAEs. Under PD-1 inhibitor combined chemotherapy, the risk of high-grade irAEs affecting critical organs and treatment-related death increases significantly, with a relative risk of 1.54 compared to chemotherapy alone (Takada et al., 2024).

This article examines a case involving a patient with non-small cell lung cancer who developed multi-system adverse reactions following the administration of immune checkpoint inhibitors, with the aim of providing insights for similar clinical scenarios.

## 2 Case information

The patient is a 74-year-old male with a height of 165 cm, a weight of 70 kg, and a body surface area of 1.75 m^2^. He was diagnosed with non-small cell lung cancer 2 months prior. Recently, he developed bilateral ptosis, which was more pronounced in the evening than in the morning, along with progressively worsening blurred vision over the past 3 days. He was admitted to the hospital on 10 March 2026, for further evaluation and treatment. Two months earlier, a routine examination revealed a thoracic mass. A subsequent chest CT scan identified a mass in the posterior segment of the right upper lung lobe, measuring approximately 4 cm × 3.1 cm, with partial obstruction of adjacent small bronchi and associated vasculature. The enhanced scan demonstrated heterogeneous enhancement, and multiple enlarged lymph nodes were observed in the mediastinum. A follow-up needle biopsy indicated squamous cell carcinoma. Immunohistochemistry results were as follows: P40 (+), CK5/6 (+), NapsinA (−), TTF (−), CD56 (−), INSM1 (−), Brg-1 (+), Vim (−), and Ki67 (approximately 40%–50%). The patient has been clinically diagnosed with non-small cell lung cancer, classified as T2N2aM0, stage IIIA. They received two cycles of neoadjuvant chemotherapy from 26 January 2026, to 22 February 2026. The treatment regimen included an intravenous administration of 240 mg of toripalimab every 3 weeks, alongside 240 mg of paclitaxel and 70 mg of lobaplatin, both administered intravenously on day one of each cycle. The patient has a decade-long history of hypertension, managed with long-term irbesartan-hydrochlorothiazide therapy, resulting in well-controlled blood pressure levels. Additionally, the patient has a 10-year history of atrial fibrillation, for which they have been receiving rivaroxaban over the past year, maintaining a stable condition. There is no significant history of infectious diseases, allergies, or familial medical conditions. Admission Diagnosis: 1. Non-small cell lung cancer (T2N2aM0 Stage IIIA); 2. Myasthenia gravis? Lambert-Eaton syndrome? Others? 3. Hypertension; 4. Atrial fibrillation.

## 3 Course of treatment

Upon admission, the patient’s vital signs were recorded as follows: temperature at 36.6 °C, pulse rate at 99 beats per minute, respiratory rate at 20 breaths per minute, and blood pressure at 132/84 mmHg. The patient was conscious and exhibited bilateral ptosis, as depicted in Figure 1. Ocular examination revealed full range of eye movement in all directions, with no evidence of nystagmus or movement disorders, and no ataxia. Pupils were equal in size, round, approximately 3 mm in diameter, and responsive to light. Limb muscle strength was assessed at grade 5, with symmetrical and normal muscle tone. Tendon reflexes were symmetrical and graded as (++), with no pathological reflexes observed. The neck was supple, and Kernig’s sign was negative. Coordination tests, including the finger-to-nose test, heel-to-knee-shin test, and Romberg sign, were negative. Auxiliary testing revealed the following thyroid function parameters: free thyroxine at 64.60 pmol/L, free triiodothyronine at 17.30 pmol/L, thyroxine at 322.80 nmol/L, and triiodothyronine at 5.47 nmol/L, all of which were significantly elevated compared to measurements taken on 22 February 2026. Thyroid-stimulating hormone (TSH) levels were measured at <0.008 mIU/L, indicating a further decrease from previous levels. The cardiac enzyme profile showed elevated levels of creatine kinase at 2651 U/L, lactate dehydrogenase at 489 U/L, and α-hydroxybutyrate dehydrogenase at 429 U/L. The cardiac protein profile reveals the following concentrations: creatine kinase MB isoenzyme at 86.2 μg/L, myoglobin exceeding 1,000.0 μg/L, and high-sensitivity troponin I at 0.1360 μg/L. The autoantibody profile testing indicates negative results for antibodies including anti-dsDNA, anti-SmD1, anti-SSA/SSB, antinuclear antibodies (ANA) via the indirect immunofluorescence (IIF) method, anti-Jo-1, anti-Mi-2, and anti-Ku. Additionally, tests for anti-myeloperoxidase antibody (MPO-ANCA), anticardiolipin antibodies IgG/M/A, anti-proteinase three antibody (PR3-ANCA), and anti-glomerular basement membrane antibody (GBM antibody) also returned negative. The levels of anti-cyclic citrullinated peptide antibody (anti-CCP), rheumatoid factor (RF), and anti-streptolysin O (ASO) are within normal limits, and the thyroid-stimulating hormone receptor antibody (TRAb) is negative. Liver and kidney function tests, along with routine blood tests, are essentially normal. The electrocardiogram (ECG) indicates the presence of atrial fibrillation and a complete right bundle branch block. Low-frequency repetitive electrical stimulation was administered to the left facial nerve, left ulnar nerve, and right accessory nerve, with no significant reduction in action potentials observed at any frequency, even following vigorous exercise. The brain MRI plain scan reveals hyperintense signals in the periventricular and deep white matter bilaterally, along with perivascular spaces in the bilateral basal ganglia and surrounding the lateral ventricles. Thyroid ultrasound findings suggest diffuse thyroid disease bilaterally. The patient presents with bilateral ptosis, which worsens in the evening, and experiences blurred vision. Initially, a neuroelectrophysiological differential diagnosis was conducted: low-frequency repetitive nerve stimulation of the left facial nerve and ulnar nerve, as well as the right accessory nerve, demonstrated no decrease in action potential amplitude, nor was there an increase in amplitude with high-frequency stimulation. Additionally, there were no characteristic signs of predominantly proximal limb weakness, effectively excluding diagnoses of myasthenia gravis and Lambert-Eaton myasthenic syndrome. Further screening for autoimmune markers, including ANA profile, ANCA, anti-CCP antibodies, rheumatoid factor, myositis-specific antibodies (such as anti-Jo-1), anti-glomerular basement membrane antibodies, and anticardiolipin antibodies, yielded negative results. The absence of rashes, joint pain, Raynaud’s phenomenon, or other features indicative of connective tissue diseases effectively excludes primary autoimmune conditions such as idiopathic inflammatory myopathy, systemic vasculitis, and rheumatoid arthritis. The patient’s thyroid function tests reveal multiple elevated indicators alongside significantly suppressed thyroid-stimulating hormone (TSH) levels, although the thyroid-stimulating hormone receptor antibody (TRAb) is negative. Ultrasound imaging of the thyroid indicates diffuse bilateral thyroid lesions. Given the observed dynamic changes in thyroid function tests before and after treatment, Graves’ hyperthyroidism can be definitively excluded. This conclusion is substantiated by an extensive assessment of the patient’s medical history, medication chronology, clinical symptoms, physical examination findings, and a comprehensive array of auxiliary test results, culminating in the final diagnosis: 1. Non-small cell lung squamous cell carcinoma (T2N2aM0, stage IIIA); 2. Immune-related myositis (extraocular muscles involved); 3. Immune-related myocarditis; 4. Immune-related thyroiditis (hyperthyroid phase); 5. Hypertension; 6. Atrial fibrillation.

**FIGURE 1:**
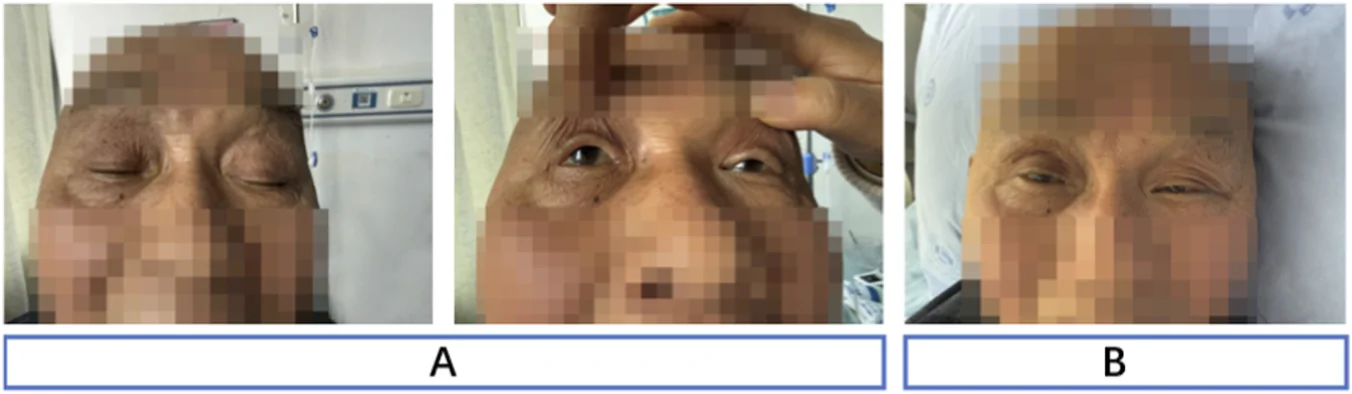
Symptom changes before and after treatment: **(A)** Before treatment: On 11 March 2026, the patient experienced worsening bilateral ptosis and difficulty opening their eyes throughout the day, accompanied by blurred vision, but without conjunctival congestion or edema; **(B)** After treatment: 18 Mar 2026: The bilateral upper eyelid ptosis significantly improved, and eye opening was better than before.

Subsequently, a comprehensive treatment regimen was implemented to address multi-system immune damage and thyroid dysfunction. The patient was diagnosed with immune checkpoint inhibitor (ICI)-related immune thyroiditis in the hyperthyroid phase and was prescribed Methimazole tablets, 20 mg orally once daily, with instructions to regularly monitor thyroid function and attend follow-up appointments. Concurrently, treatment for autoimmune myocarditis combined with autoimmune myositis was initiated with 80 mg of methylprednisolone sodium succinate administered intravenously once daily. After 7 days of steroid therapy, the patient’s ocular symptoms exhibited significant improvement, as illustrated in Figure 1, and related clinical indicators showed marked enhancement compared to baseline values. Upon the patient’s request for discharge, the treatment regimen was adjusted to 40 mg of oral prednisone acetate tablets once daily, with a recommendation to gradually taper the dosage. During a follow-up visit on 8 April 2026, the patient’s clinical indicators had largely normalized; however, the hyperthyroidism had progressed to hypothyroidism, necessitating the administration of 25 μg of levothyroxine daily. Subsequently, the patient underwent a right upper lobe lung resection on 13 April 2026. The changes in key indicators during the patient’s treatment process and the treatment process are shown in Figure 2.

**FIGURE 2:**
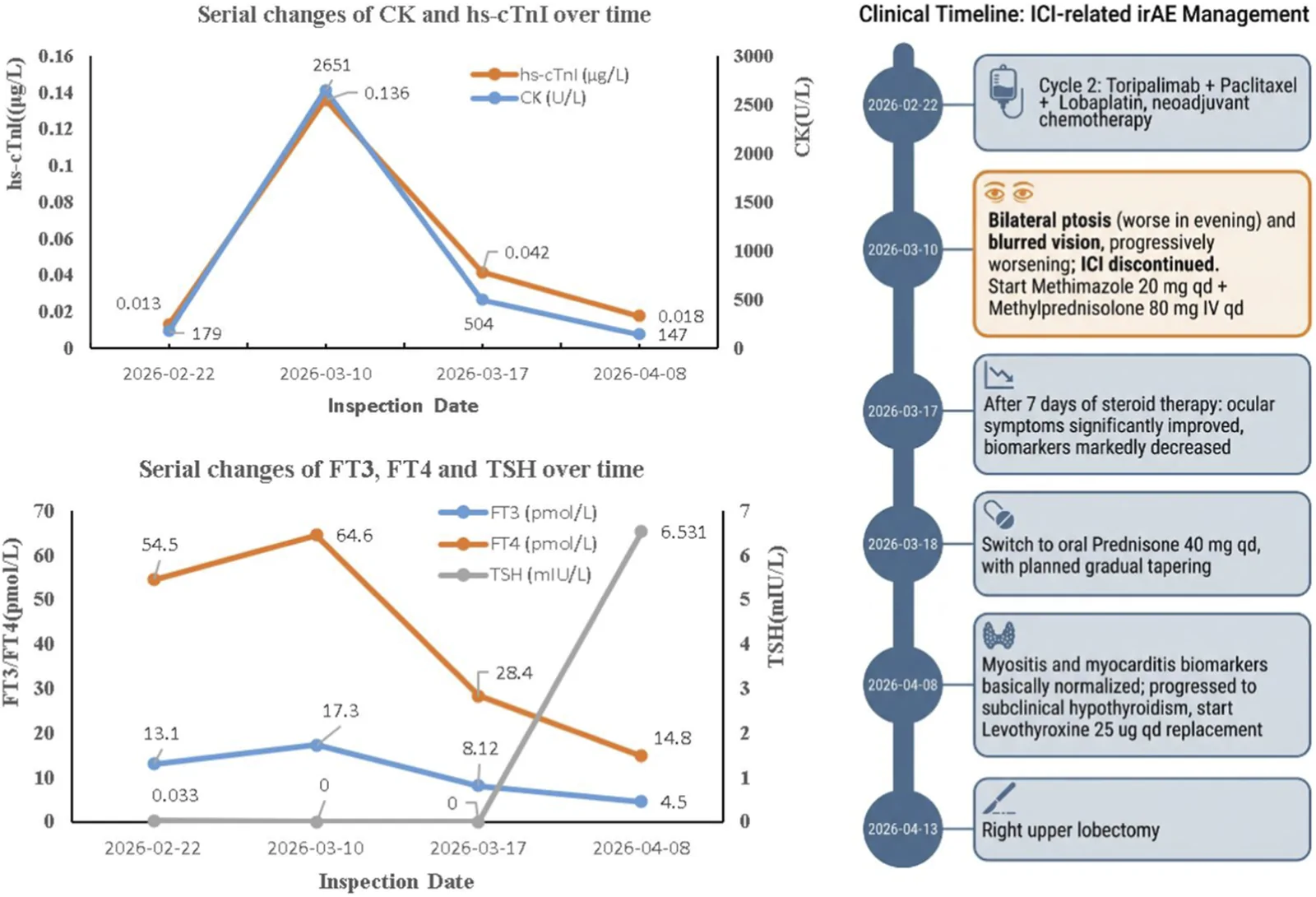
Biomarker trends and clinical timeline for ICI-related adverse events.

## 4 Discussion

Programmed cell death protein 1 (PD-1) interacts with its ligand, programmed death-ligand 1 (PD-L1), which is expressed on the cell membranes of antigen-presenting cells (APCs) and tumor cells. Inhibition of either PD-1 or PD-L1 can enhance T cell activity and promote anti-tumor immunity; however, it may also disrupt peripheral self-tolerance mechanisms, potentially precipitating the onset or exacerbation of autoimmune diseases (O'Hare et al., 2024). This report examines a 74-year-old patient with a history of chronic cardiovascular conditions who was undergoing treatment with a combination of PD-1 inhibitors and dual chemotherapy. The NEOTORCH III study (Fang et al., 2026)showed that using toripalimab with paclitaxel and cisplatin around surgery lowers the cancellation rate from 26.7% to 17.8%. Tumor and lymph node downstaging rates were 80.7% and 67.5%, respectively, leading to a 50% reduction in disease progression or death risk. This regimen does not increase surgical difficulty or complications and is safe. Before starting this treatment, the patient had no history of active autoimmune diseases. Their blood pressure and atrial fibrillation are well-managed with medication, and they have no history of severe heart failure or myocardial infarction. With no drug allergies and a suitable physical condition, they can undergo neoadjuvant chemoimmunotherapy for stage IIIA lung squamous cell carcinoma, potentially allowing for surgical removal. However, previous studies have indicated that individuals over the age of 60 and those with pre-existing autoimmune diseases are at an increased risk of experiencing severe immune-related adverse events (irAEs) (Fang et al., 2025; Li et al., 2025). Research suggests that PD-1 inhibition may result in aberrant distribution of cardiac collagen through interactions between endothelial cells and fibroblasts, as well as through endothelial-to-mesenchymal transition (EndMT) (Zhang et al., 2025). A meta-analysis has also identified an association between the use of immune checkpoint inhibitors and cardiotoxicity (Rubio-Infante et al., 2021). These findings imply that PD-1 inhibitors may elevate the risk of cardiovascular side effects in patients with pre-existing cardiovascular conditions. The findings suggest that physicians should exercise heightened caution when administering PD-1 inhibitors in conjunction with dual chemotherapy in patients with multiple comorbidities, particularly those with a history of cardiovascular disease. Chemotherapy has the potential to further activate the immune system, thereby increasing the risk of autoimmune damage across multiple organs. Research indicates that chemotherapeutic agents can enhance immune responses by modulating the types and functions of immune cells within the tumor immune microenvironment. For instance, agents such as cisplatin have been shown to augment anti-tumor immunity when used in combination with immune checkpoint inhibitors. This combination not only enhances the efficacy of chemotherapy but also reduces the prevalence of immunosuppressive cells, such as myeloid-derived suppressor cells (MDSCs) and regulatory T cells (Tregs). Nonetheless, the integration of chemotherapy with immunotherapy also elevates the risk of immune-related adverse events (irAEs) (Fang et al., 2025; Barnestein et al., 2022; Li et al., 2022; Ma et al., 2023).

Immune activation induced by immune checkpoint inhibitor therapy can impact any tissue, resulting in a spectrum of clinical manifestations. The onset of immune-related adverse events (irAEs) exhibits considerable interindividual variability, occurring from a few days following the initial dose to several months post-final dose. Nevertheless, irAEs most frequently manifest within the first 3 months of immune checkpoint inhibitor treatment. In this particular case, the patient initially developed immune-related thyroiditis following one cycle of medication, whereas adverse effects involving the peripheral nervous system and cardiac tissue emerged after two cycles. This observation underscores that, even within the same patient, the temporal occurrence of side effects can differ across various organ systems.

Neurological immune-related adverse events (n-irAEs) represent infrequent yet high-risk complications associated with immune checkpoint inhibitor (ICI) therapy, manifesting in approximately 1%–2% of cases. Severe n-irAEs occur in fewer than 0.5% of patients, predominantly affecting the neuromuscular system and/or peripheral nerves. Notable serious neurological irAEs include immune-related myositis, polyradiculoneuropathy, and encephalitis (O'Hare et al., 2024; Picca and Psimaras, 2026). The likelihood of chronic irAEs within the nervous system is substantial, with over 50% of patients developing chronic conditions following the acute phase, underscoring the critical importance of long-term management strategies (Picca and Psimaras, 2026; Rossi et al., 2024). In this particular patient, early recognition and timely intervention of immune myositis, primarily affecting the ocular muscles, successfully averted any persistent neurological complications. Among patients undergoing checkpoint inhibitor therapy, 42% of fatalities are attributed to immune-related adverse events involving the nervous system and/or cardiac complications. Myositis, as an irAE induced by ICIs, frequently co-occurs with myocarditis and myasthenia gravis, collectively referred to as the ‘triple syndrome.’ The management of this syndrome is complex and is associated with a high mortality rate (O'Hare et al., 2024; Stavropoulou De Lorenzo et al., 2024; Rubino et al., 2026). In contrast to the typical triad observed in myasthenia gravis with overlapping syndromes, this patient exhibited a combination of immune-mediated myositis and immune-mediated myocarditis. Despite the absence of myasthenia gravis involvement, the risk of severe deterioration remained significantly high. Regarding treatment, the management strategies for immune checkpoint inhibitor (ICI)-induced myocarditis and myositis are largely similar: immediate discontinuation of ICIs and initiation of high-dose corticosteroids as the first-line therapy are recommended (Mauriello et al., 2026). For cases where corticosteroids prove ineffective, second-line therapeutic options include mycophenolate mofetil (MMF), intravenous immunoglobulin (IVIG), and emerging therapies such as abatacept and ruxolitinib (Mauriello et al., 2026; Moyambi et al., 2026). Furthermore, research has identified the critical role of CXCR6 T cells in the pathogenesis of ICI-related myocarditis, suggesting that therapies targeting CXCR6 may represent a future treatment strategy. Rituximab, a B-cell targeted therapy, has shown promise in managing certain refractory immune-related adverse events (irAEs), particularly when conventional immunosuppressive treatments are unsuccessful (Hermabessiere et al., 2026). In this instance, the patient experienced a favorable outcome, with no enduring cardiac or neurological damage, underscoring the critical importance of early identification of overlapping multi-system immune-related adverse events (irAEs), prompt discontinuation of the offending drug, and the rapid initiation of immunosuppressive therapy. Negative serum autoantibodies cannot exclude ICI-mediated myositis/myocarditis. Diagnosis should rely on clinical signs, muscle enzymes, troponin, ECG, and ICI exposure timeline (Gonçalves et al., 2025; Suhan et al., 2025). Our patient developed ICI-related myositis despite negative autoantibodies, highlighting that these tests should not rule out immune-related toxicities. Clinicians should remain alert for ICI-associated myositis and myocarditis in patients with muscle weakness, ptosis, or elevated cardiac biomarkers, even if autoantibody tests are negative, to prevent missed diagnoses of overlapping multi-system irAEs.

Thyroiditis induced by immune checkpoint inhibitors (ICIs) represents one of the more prevalent endocrine side effects. Research indicates that the incidence of thyroid dysfunction during ICI treatment is approximately 2.2%, with a median onset time ranging from 8 to 12 weeks following the initiation of ICI therapy (Jafry et al., 2026; Husebye et al., 2022). In this particular case, the patient developed thyroiditis within 3 weeks of commencing the medication, and within 10 weeks, the condition progressed from hyperthyroidism to hypothyroidism. Most patients with ICI-related thyroiditis are asymptomatic or present with only nonspecific symptoms such as fatigue and lethargy, typically detected through routine thyroid function assessments (Li Jiarong, 2019). However, elevated thyroid hormone levels can exacerbate a patient’s myositis symptoms (Tee et al., 2020; Nakao et al., 2020). The patient has elevated thyroid hormones and decreased TSH. Thyroid ultrasound shows diffuse lesions, and TSH receptor antibodies are negative, ruling out Graves’ disease. This fits the characteristics of ICI-related autoimmune thyroiditis, which typically starts with hyperthyroidism and gradually progresses to hypothyroidism. In this case, the patient’s later thyroid function changes also confirmed this pattern. Thyroiditis caused by ICIs is basically a destructive thyroiditis. The hyperthyroid phase happens because the thyroid follicular cells are damaged by the immune system, causing stored thyroid hormones to be passively released into the blood, rather than the thyroid overproducing hormones itself. So, anti-thyroid drugs like methimazole, which work by blocking hormone production, do not really help here. They’re only recommended for 6–8 weeks or if Graves’ disease is also present. Specific handling principles (Cui and Li, 2023; Zhu et al., 2025; Schneider and Kaffenberger, 2026):1. The primary focus should be on supportive treatment aimed at symptom control. 2. Beta-blockers are typically the first-line therapy for managing symptoms such as palpitations and tremors. However, some cases have demonstrated that patients with severe immune checkpoint inhibitor (ICI)-related thyroiditis have achieved more favorable outcomes with methimazole, or even when combined with corticosteroids (Fasano et al., 2022; Huo et al., 2023). In February 2026, when this patient was evaluated for thyroid abnormalities, they were not on methimazole. Upon admission, the patient’s free triiodothyronine (FT3) and free thyroxine (FT4) levels were significantly elevated, while thyroid-stimulating hormone (TSH) was markedly suppressed at less than 0.008 mIU/L, indicating severe grade 3 hyperthyroidism. Additionally, the patient suffers from autoimmune myositis concurrent with hyperthyroidism, resulting in a state of heightened metabolic damage to the muscles. The presence of atrial fibrillation and complete right bundle branch block further complicates the clinical picture, as hyperthyroidism can exacerbate arrhythmias and increase myocardial oxygen consumption, thereby elevating the patient’s cardiovascular risk. In this instance, we are administering a short-term, low-dose regimen of methimazole to regulate thyroid hormone levels and alleviate the elevated metabolic burden. Approximately 75% of patients with similar conditions may transition to hypothyroidism following the hyperthyroid phase, with the median time for this transition being 4–7 weeks, at which point levothyroxine replacement therapy may become necessary (Chaker et al., 2024; Muir et al., 2020; Zhang et al., 2023). On 8 April 2026, the patient underwent a follow-up thyroid function test, which revealed that FT3 and FT4 levels had normalized, while TSH levels had increased from significantly suppressed to elevated (6.531 mIU/L). This indicates that the immune checkpoint inhibitor (ICI)-related autoimmune thyroiditis has progressed from the hyperthyroid phase to the subclinical hypothyroid phase. Consequently, the patient is now receiving 25 μg of levothyroxine daily as replacement therapy.

The 2025 Asian Consensus advises a 4–6 weeks gap between completing neoadjuvant immunotherapy (with chemotherapy) and surgery for non-small cell lung cancer (Saw et al., 2025). However, severe immune-related adverse events (irAEs) often delay surgery. A study (Gandhi et al., 2026) of 87 patients with severe irAEs from neoadjuvant or adjuvant ICIs found that 27.3% of those in the neoadjuvant group faced surgery delays due to irAEs, with a median delay of 21 days. Grade ≥3 irAEs pose severe risks, making surgery under these conditions highly risky. Surgery should be delayed if preoperative irAEs are grade ≥ G3, until toxicity reduces to ≤ G1 and the patient stabilizes. During this period, assess irAEs biweekly and evaluate tumor resectability every 4–6 weeks to adjust the treatment plan and determine the best surgery timing (Saw et al., 2025). After neoadjuvant immunotherapy, ensure normal thyroid (FT3, FT4) and adrenal function, and recovery of myocarditis, pneumonitis, hepatitis, nephritis, and skin toxicity to grade 1. Focus on screening for myocarditis, endocrine disorders, and pneumonitis, and tailor surgery timing with a multidisciplinary team (Saw et al., 2025; Gandhi et al., 2026; Gastric Cancer Professional Committee, China Anti-Cancer Association, 2024). In this instance, a lobectomy was conducted once the clinical symptoms and laboratory indicators associated with myositis and myocarditis had subsided to grade 1 or lower, and thyroid toxicity had stabilized following appropriate intervention. The patient demonstrated a favorable postoperative recovery.

## 5 Summary

The combination of PD-1 inhibitors and chemotherapy can induce overlapping immune-related adverse events (irAEs) across multiple organ systems, characterized by complex clinical manifestations and organ heterogeneity. In instances where patients exhibit symptoms such as ptosis or muscle weakness following treatment, clinicians should not solely concentrate on excluding neuromuscular junction disorders. Comprehensive diagnostic evaluations, including assessments of muscle enzyme levels and cardiac biomarkers, are imperative to identify potential underlying multi-organ irAEs. Even if clinical manifestations are confined to a single system, vigilance for immune-mediated damage in other organs is essential. In cases of concurrent inflammatory myopathy and myocarditis, it is crucial to promptly discontinue immune therapy and initiate appropriate glucocorticoid therapy to mitigate excessive immune activation. The progression of endocrine irAEs is dynamic; thus, treatment strategies should be tailored to the patient’s specific stage, whether hyperthyroid or hypothyroid. Establishing a systematic follow-up protocol to monitor organ function over time is vital for reducing the risk of severe immune toxicity and enhancing patient outcomes.

## Data Availability

The original contributions presented in the study are included in the article/supplementary material, further inquiries can be directed to the corresponding author.
